# Texas State Medical Association

**Published:** 1894-03

**Authors:** 


### Society Notes.

### TEXAS STATE MEDICAL ASSOCIATION.

The officers of the following Sections have written the JOURNAL, requesting us to publish a call for papers for their respective Sections. Those who intend to contribute papers, should send the title to the Secretary of the Section under which the paper comes, by April 1st or earlier, so that a place in the program may be assigned each paper, otherwise papers may be excluded for want of opportunity to read them:

Section on Gynecology—

Dr. G. W. Christian, Chairman, Houston.
Dr. R. W. Knox, Secretary, Houston.

Section on Surgery—

Dr. C. A. Smith, Chairman, Tyler.
Dr. Clay Johnson, Secretary, Corsicana.
(Dr. Johnson is absent; send papers or title to Dr. Smith.)

Section on Medical Jurisprudence, Chemistry and Psychology—

Dr. B. F. Brittain, Chairman, Baird.
Dr. H. M. Cravens, Secretary, Arlington.

Section on State Medicine and Public Hygiene—

Dr. T. J. Bennett, Chairman, Austin.
Dr. V. P. Armstrong, Secretary, Dallas.

For the information of our readers and those who wish to contribute to the program, we give the officers of the other Sections:

Section on Practice of Medicine—

Dr. J. C. Jones, Chairman, Gonzales.
Dr. I. E. Clarke, Secretary, Schulenburg.

Section on Obstetrics and Diseases of Women—

Dr. E. Randall, Chairman, Galveston.
Dr. A. B. Gardner, Secretary, Belleville.

Section on Ophthalmology and Otology—

Dr. R. H. Chilton, Chairman, Dallas.
Dr. T. J. Tyner, Secretary, Austin.

Section on Dermatology, etc.—

Dr. F. E. Daniel, Chairman, Austin.
Dr. S. E. Hudson, Secretary, Austin.

Section on Microscopy and Pathology—

Dr. A. J. Smith, Chairman, Galveston.
Dr. D. Cerna, Secretary, Galveston.

